# Influence of clerks’ personality on their burnout in the clinical workplace: a longitudinal observation

**DOI:** 10.1186/s12909-016-0553-0

**Published:** 2016-01-28

**Authors:** Cheng-Chieh Lin, Blossom Yen-Ju Lin, Chia-Der Lin

**Affiliations:** School of Medicine, China Medical University, No.91, Hsueh-Shih Road, Taichung, 404 Taiwan ROC; Department of Family Medicine, China Medical University Hospital, No.2, Yude Road, Taichung, 404 Taiwan ROC; Department of Healthcare Administration, College of Medical and Health Science, Asia University, No. 500, Lioufeng Rd., Wufeng, Taichung, 413 Taiwan ROC; Department of Education, Department of Otolaryngology, China Medical University Hospital, No.2, Yude Road, Taichung, 404 Taiwan ROC

**Keywords:** Clerks, Medical students, Burnout, Big Five personality traits, Emotional stability, Openness

## Abstract

**Background:**

The clinical training of medical students in clerkship is crucial to their future practice in healthcare services. This study investigates burnout during a 2-year clerkship training period as well as the role of personality traits on burnout during training.

**Methods:**

Ninety-four clerks at a tertiary medical centre who provided at least 10 responses to a routine survey on clinical rotation were included in this study, which spanned September 2013 to April 2015. Web-based, validated, structured, self-administered questionnaires were used to evaluate the clerks’ personalities at the beginning of the first clerkship year, and regular surveys were conducted to evaluate their burnout at each clinical specialty rotation throughout the 2-year clerkship period. Overall, 2230 responses were analysed, and linear mixed-effects models were used to examine the repeated measures of the clerks.

**Results:**

Our findings revealed that medical student burnout scores were lower in the second year than they were in the first year of clerkships. Using the Big Five personality factors, all of the propensities, namely extroversion, agreeableness, consciousness, emotional stability, and openness were related to different extents of burnout reduction in the first clerkship year (*P* < .05). However, only emotional stability and openness were related to clerks’ reduced burnout in the second clerkship year. Furthermore, being female, older, and with accompanied living were more closely related to lower burnout compared with being male, younger, and living alone throughout the clerkship period.

**Conclusions:**

The students in the first-year clerkship, particularly those with higher burnout levels, had tendencies in the Big Five personality characteristics, exhibiting higher levels of introversion, antagonism, lack of direction, neuroticism, and not open to new experiences. The students in the second-year clerkship who do not exhibit a high propensity for emotional stability and openness should be of particular concern. The findings can serve as a reference for clinical teachers and mentors to effectively prevent and reduce the burnout of medical students during clerkship training at clinical workplaces.

## Background

“Burnout” refers to a psychological syndrome involving chronic emotional and interpersonal stressors that individuals experience in specific environments, which might subsequently affect responses to their tasks, organizations, co-workers and peers, clients, and themselves [1]. Literature reviews have revealed that burnout is prevalent in medical students, and may persist beyond medical school [2]. In major US multi-institutional studies, at least half of all medical students have been estimated to suffer from burnout [2], and medical students, residents, fellows, and emergency care physicians were more predisposed to burnout compared with the general US population [3]. A prospective cohort study found that for medical students, burnout is related to a high intention to drop out of medical school [4], and an increased likelihood of subsequent suicidal ideation [5]. Burnout is also related to health impairment [6] and low job performance [7]. Burnout in the workplaces and their antecedents have been noted to be typically categorized as organizational, professional characteristics, and individual [8, 9].

The clinical training stage is a crucial transition period for medical students because it represents the time when students’ roles substantially change from student to physician; clerks must bear a great social responsibility, despite lacking medical licences. The clinical training stage presents vigorous challenges related to the intelligence, physical strength, and stamina of medical students [10–12]. The transition from preclinical to clinical training has been identified as a crucial stage of medical school for student stress [13]. The effects of personality on burnout have been studied for several reasons [14–16]. For instance, people with certain personality traits might self-select out of highly stressful and burnout-conducive occupations and jobs [17]. Another reason is that certain personality traits may predispose people to stressors, subsequently eliciting burnout [14]. Several previous empirical studies have focused on the role of personality in medical student burnout. In a 3-year prospective questionnaire- and interview-based study conducted by Dahlin and Runeson in Sweden [18], high burnout was predicted for 127 first-year medical students whose cases were followed until their third (clinical training) year because of their impulsivity trait (the negatively oriented side of conscientiousness). Gramstad, Gjestad, and Haver studied third-year medical students in Norway, and found that neuroticism and reality weakness are related to high levels of perceived job stress and higher levels of anxiety and depression symptoms during an internship [19]. The awareness of personality traits may constitute opportunities versus risks in managing circumstances. However, the cross-sectional exploration of the effects of personality on medical students might not trace the changes in their burnout levels, and may be unable to predict these effects in the future [18–20]. Moreover, the cultural constitution of personality differs between Western and non-Western countries, and how personality traits are related to culturally appropriate behavioural outcomes may vary based on a different sociocultural world [21, 22]. The experience of burnout and the roles of personality traits may differ in an Asian ﴾Chinese﴿ context compared with what is known of burnout in Western cultures. Hence, this study investigates burnout throughout the 2-year clerk training period for medical students in Taiwan, and how their personality traits affect burnout during clerkship over time.

## Methods

This prospective cohort Web-based questionnaire study was ethically approved by the Research Ethics Committee of China Medical University and Hospital [CMUH102-REC3-088 and CMUH102-REC3-088(CR-1)]. All the participants provided written statements detailing consent for participation.

### Participants and data collection

Our study comprised one cohort of fifth- and sixth-year medical students in a 7-year medical education programme at China Medical University, Taiwan, as clerks between September 2013 and April 2015. Of the 190 clerks who attended recruitment meetings, 111 (58.4 %) agreed to participate in the study and provided written informed consent. The participants first completed a validated, structured, self-administered questionnaire describing their personality and personal background at the beginning of the clerkship in September 2013. They subsequently, through April 2015, regularly answered Web-based validated, structured, self-administered questionnaires that evaluated their burnout at each specialty rotation. Furthermore, all participants were free to decide whether to complete the survey each time, and each participant presented various responses during the study period. Participants with at least 10 responses to our routine study survey, indicating their longitudinal participation for at least 6 months, were included in this study. Overall, 94 clerks who provided 2230 responses (1351 and 864 responses during the first and second clerkship years, respectively) were included in our analysis.

### Study development and administration

#### Participant personality

The Big Five personality inventory is widely applied in medical education studies. The included traits were measured using the Big Five Inventory, which measures the traits of extraversion, agreeableness, conscientiousness, neuroticism (reversed as ‘emotion stability’), and openness [23]. Extraversion refers to a person’s capability for joy and the tendency to seek interpersonal relationships, symbolising the traits of socialisation, dominance, energy, and positive effects. The agreeableness of a person involves helping others and behaving prosocially (ie, being cooperative, nurturing, affectionate, sensitive, caring, altruistic, kind, tender, and soft-hearted). Conscientiousness is viewed as a motivational trait, and refers to striving for achievement, prudence, dependability, persistence, order, and impulse control. Neuroticism often involves anxiety, depression, anger, embarrassment, worry, fearfulness, instability, and insecurity. Openness refers to influencing the breadth and complexity of individuals’ mental experiences, symbolising imagination, curiosity, originality, broadmindedness, and intelligence [24]. To reduce the time required for answering questionnaires, a brief 10-item Big Five personality inventory was developed and validated by Gosling, Rentfrow, and Swann [25]; this inventory has been adopted in medical education studies [26, 27]. This brief personality inventory with a 5-point scale was used in the current study. To unify the tendency of personality in the same direction (positive orientation), the neuroticism score was reversed to obtain the emotional stability score.

#### Burnout for each clinical specialty rotation

In our study, participant burnout was measured using the Professional Quality of Life: Compassion Satisfaction and Fatigue Version 5 scale [28]. This scale covers several constructs, such as compassion satisfaction, burnout, and secondary traumatic stress [28], and has been gradually adopted in medical studies [29–31]. Burnout is a negative emotion associated with feelings of hopelessness and difficulty in managing work or performing a job effectively, and was measured using 10 items answered on a 5-point scale for our study [32]. The 10 items enquired how frequently participants experienced various emotions, and the responses were 1 (*never*), 2 (*seldom*), 3 (*sometimes*), 4 (*often*), and 5 (*always*). The Cronbach alpha value was 0.770. The burnout score calculation comprises the following steps: (1) reversing the necessary items for burnout, and (2) summing the items for burnout [32]. The summed score was used for further analysis. Participant burnout was regularly measured after each clinical specialty rotation during clerkship training from September 2013 to April 2015.

#### Participant characteristics

In this study, demographic data included gender, age, and living status (alone or accompanied), all of which might be associated with burnout [31, 33, 34].

### Statistical analysis

Descriptive analyses were performed to examine participant personality at baseline and burnout at various clinical specialty rotations, as perceived by them. Because each participant was not an independent cohort (ie, their responses were treated as correlated data), did not exhibit unequal variance, and involved an unequal number of repetitions, linear mixed-effects models were employed during both clerkship training years [35, 36]. In each year, individual participant burnout, personality, and demographic characteristics were used as dependent variables, independent variables, and covariates, respectively. All analyses were performed using SPSS (version 20.0).

## Results

Ninety-four participants (49 men (52 %), 45 women (48 %); average age: 23 y) were included in our study. Among them, 31 participants (33 %) lived alone, and 62 (66 %) were nonreligious. Table 1 lists the detailed personal information.

**Table 1:** Personal characteristics of participants (*n* = 94)

Variables	Mean	SD	Frequency	%
Age	23.38	2.42		
Gender				
Male			49	52
Female			45	48
Living status				
Alone			31	33
Accompanied			63	67
Religion				
No			62	66
Yes			32	34
Personality				
Extroversion	3.07	0.88		
Agreeableness	3.86	0.56		
Consciousness	3.45	0.75		
Emotional Stability	3.26	0.85		
Openness	3.63	0.70		
Burnout^*^				
First-year clerkship	24.35	5.11		
Second-year clerkship	23.55	5.57		

^*^Significant difference (*P* < .05) was observed between the first- and second-year clerkship students examined using mixed modelling that accounted for repeated measures of individual participant burnout

We analysed 2230 responses obtained from all of the participants. The mean participant burnout scores were 24.35 and 23.55 for students in their first and second clerkship years, respectively. Scores ranged from 10 to 50; a higher score indicated greater burnout. The linear mixed-effects model, which used the repeated measures of burnout as a dependent variable and clerkship year as an independent variable, revealed that participant burnout scores were lower in the second year than they were in the first year (*P* = .001).

In the first clerkship year (Table 2), considering the age, gender, and living status as covariates, the linear mixed-effects model revealed that extroversion, agreeableness, consciousness, emotional stability, and openness were related to different extents of burnout reduction during clerkship (*P* < .05). Being male, younger, and living alone were related to increased burnout.

**Table 2:** Effect of participant personality on their burnout at clinical workplaces during the first clerkship year

Parameters	Estimates	SE	*P* value	95 % confidence interval	
				Lower	Upper
Big-Five personality					
Extroversion	-0.378	0.168	0.025	-0.709	-0.048
Agreeableness	-0.843	0.248	0.001	-1.330	-0.356
Consciousness	-0.431	0.187	0.022	-0.799	-0.064
Emotional Stability	-0.674	0.182	0.000	-1.031	-0.318
Openness	-0.777	0.207	0.000	-1.183	-0.371
Personal characteristics					
Age	-0.250	0.055	0.000	-0.357	-0.143
Gender (default: female)	0.583	0.277	0.035	0.040	1.125
Living status (default: accompanied)	1.796	0.280	0.000	1.248	2.345

In the second clerkship year (Table 3), considering the age, gender, and living status as covariates, the linear mixed-effects model revealed that only emotional stability and openness were related to reduced burnout during clerkship (*P* < .05). Being male, younger, and living alone were related to increased burnout.

**Table 3:** Effect of participant personality on their burnout at clinical workplaces during the second clerkship year

Parameters	Estimates	SE	*P* value	95 % confidence interval	
				Lower	Upper
Big-Five personality					
Extroversion	0.431	0.244	0.078	-0.049	0.910
Agreeableness	-0.115	0.347	0.742	-0.797	0.568
Consciousness	-0.114	0.251	0.651	-0.608	0.380
Emotional Stability	-0.547	0.274	0.046	-1.085	-0.009
Openness	-1.126	0.297	0.000	-1.709	-0.543
Personal characteristics					
Age	-0.507	0.077	0.000	-0.658	-0.356
Gender (default: female)	0.845	0.371	0.023	0.116	1.574
Living status (default: accompanied)	2.367	0.377	0.000	1.626	3.107

## Discussion

Medical students undergoing clinical training experience high stress because they are expected to enhance their clinical knowledge and skills in actual clinical workplaces while interacting with patients [10, 37], thus contributing to considerable workplace difficulties [38]. The 2230 responses obtained in this study were analysed to determine the effects of personality on participant burnout during clinical workplace training. Our findings revealed that participant burnout scores were lower in the second year than they were in the first year of clerkships. In keeping with the Big Five personality factors, all of the propensities (extroversion, agreeableness, consciousness, emotional stability, and openness) were related to different extents of burnout reduction in the first clerkship year (*P* < .05). However, only emotional stability and openness were related to the clerks’ reduced burnout in the second clerkship year. Furthermore, being female, older, and with accompanied living were more closely related to lower burnout compared with being male, younger, and living alone throughout the clerkship period.

### Burnout across clerkship years

Our findings revealed that medical students experience higher burnout during the first clerkship year than they do during the second clerkship year. In a similar manner, Santen et al reported a moderate or high burnout degree in 43 % of third-year medical students and 31 % of fourth-year medical students in the clinical workplaces (*P* < .05) in the US [39]. During the longitudinal clerkship, the first-year clerkship students were undergoing a transition and entering an unfamiliar area. Lacking advance warnings regarding the changes, first-year clerkship students experienced moments of confusion and burnout [40]. This might result in first-year clerkship students enduring first-contact shock from the transition of academic learning to real clinical scenarios, and thus, having higher burnout than during their second-year clerkships. In addition to the preclinical knowledge and skill curricula for first-year clerkship students, preventive programs might be initiated early in the preclinical school years or early and continuously in the clinical training stages such as programs for reducing stress and fostering self-awareness [41, 42]; programs to reduce depression and suicide risk [43]; wellness programs about physical activity, health, happiness, and quality of life [44, 45]; behavioural interventions designed to teach self-regulatory skills for stress reduction and emotion management [46]; and even leisure and recreational gathering hours for peer discussions and sharing.

### Personality on burnout in first-year clerkship

Using the Big Five personality traits as categories, extroversion, agreeableness, consciousness, emotional stability, and openness were related to various extents of burnout reduction during the first clerkship year (*P* < .05). Because all the traits affect medical students’ burnout, we must understand how various Big Five personality traits affect burnout through different mechanisms. For example, ‘extraversion’ refers to spending more time socialising in learning environments [47], and might be effective in settings requiring interpersonal interaction [48], such as clinical years of medical education requiring greater interactions with both patients and colleagues [24]. Gramstad, Gjestad, and Haver [19] studied third-year medical students in Norway, and also found that extroversion protected against symptoms of depression. ‘Agreeableness’ is considered empathy for how others think and feel, and is related to the clinical performance of medical residents [49]. Medical students’ empathy in the context of patient care was confirmed to be associated with positive personality characteristics conducive to relationship building [50]. Training in mindfulness, self-reflection, and emotion skills was verified to help medical students and professionals recognize, regulate, and behaviourally demonstrate empathy within clinical and professional encounters [51]. ‘Conscientiousness’ has been found to predict work-related outcomes, such as professional success, and represents adaptive coping strategies [52], and is therefore likely to influence the availability and use of coping resources to address work-related demands [53] and reduce the possibility of burnout [54] for clerks in clinical training. ‘Neuroticism’*,* a reversed personality trait of emotional stability, has been characterised as anxious, insecure, depressed, fearful, and nervous [55–57], and therefore, causes negative health outcomes, such as depression [58] and burnout [1, 59]. ‘Openness’ is considered a trait relevant for adequately adapting to novel and unforeseen changes [60], circumstances more likely to occur during clinical training years. From a practical perspective, mentors and clinical teachers may propose certain interventions through the mechanisms mentioned to assist or enhance clerks’ adaptation to clinical workplace training.

### Personality on burnout in second–year clerkship

Only emotional stability and openness were related to decreased participant burnout in the second clerkship year. Individuals high in neuroticism are likely to focus on the negative aspects of a situation [61], and are therefore more likely to encode and recall negative information from a situation afterwards [62, 63]; negative events during first-year clerkship might be cumulative to the second-year clerkship. Specific burnout reduction programs might be designed for medical students in clerkships, especially in second-year clerkships, for those with high neuroticism. In addition to the awareness of medical students with high neuroticism, clinical mentors or teachers might have to identify various personal and professional stressors (events) experienced negatively during training [64–66]. In addition, self-care skills might be equipped for medical students to assess their personal distress, recognize what they need, and develop strategies to promote their wellbeing [67, 68].

Individuals with a high degree of openness to experience tend to be more intellectually curious and open-minded about their environments [55, 69]. Open individuals do not experience feelings of uneasiness and apprehension regarding future work conditions involving uncertainty or ambiguity [1]. Open medical students viewed the new challenges of rotating clinical specialties in their second-year clerkships not as a lack of achievement or competence, but as opportunities for personal growth [70].

### Implication: what we can do for personality changes for clerk adaption in the clinical workplaces

Although the Big Five personality traits are stable as basic tendencies, scholars have argued that people can change their patterns of behaviours, thoughts, and feelings for environment adaptations [71, 72]. Several mechanisms might be useful for clerks in promoting personality changes from sociology perspective and behavioural and role theories [73], including recognising the role demands, expectations, and conflicts faced by clerks in the preclinical curriculum or orientation training; reflecting on the actions of clerks in the clinical workplaces; observational learning; and receiving feedback from clinical mentors, clinical teachers, colleagues, and peers. We recommend further study in more medical schools or teaching hospitals to understand the personality of clerks, particularly easily identifiable traits of the Big-Five personality by using 10-item brief personality test. Our findings may provide clinical teachers or mentors with indications to more effectively prevent and reduce medical student burnout during clerkships at clinical workplaces.

### Other factors affecting burnouts in clerkships

Being female, older, and with accompanied living were related to lower burnout compared with being male, younger, and living alone throughout the first and second clerkship years. The effect of sex of medical students on burnout appeared to be inconclusive in previous studies. In a meta-analysis on multiple professions, Purvanova and Muros rejected the notion that burnout is more commonly experienced by female employees [34], and that male medical students appear more susceptible to depersonalisation [74]. In accordance with the current study, higher burnout levels have been reported among younger physicians [75, 76]. Older and experienced students may be more mature, and thus, may more quickly adapt to clinical conditions compared with younger students. Moreover, in this study, participants living with friends, peers, or family were less predisposed to burnout, possibly because of social identification, which emphasises the benefit of strong connections within a group [77]. Living companions, whether family members or friends, can provide social support and thus help medical students release stress or ease distress accumulated in the workplace, particularly when transitioning from their roles as medical students in schools to clerks in clinical workplaces.

### Limitations and future work

This study has several limitations. First, we measured clerks’ personality at the beginning of clerkship and assumed no change during the 2 years of clerkship; similar assumptions have been reported regarding the stability of personality [18, 19, 24]. However, studies on adult personality development have indicated that, despite no noticeable changes in the mean level of all Big Five factors from adolescence until approximately 30 years of age [72], neuroticism, extraversion, and openness decline thereafter, whereas agreeableness and consciousness improved. Therefore, from a practical perspective, mentors and clinical teachers may require a detailed understanding of clerks’ personality changes to provide them with suitable support. Longitudinal examinations continuing through the internship and postgraduate residency years can be conducted in the future. Moreover, the responses obtained in this study might not represent all clerks nationally, limiting the generalisability of the results. Person-environment fit theory [78, 79] might be examined for more recruited medical students from more than one hospital to further explore the interactions between individuals’ personalities and specific environmental factors (eg, different hospital cultures or climates) and the connection to workplace burnout.

## Conclusion

Using a prospective study design, we examined a cohort of medical students during a 2-year clerkship programme. We discovered that medical student burnout scores were lower in the second year than they were in the first year of clerkships. First-year clerkship students who exhibited a tendency in the Big Five personality traits (ie, introversion, antagonism, lack of direction, neuroticism, and not being open to experiences) as well as second-year clerkship students who did not reveal a high propensity of emotional stability and openness warrant attention.
